# Peroxide-Dependent Analyte Conversion by the Heme Prosthetic Group, the Heme Peptide “Microperoxidase-11” and Cytochrome c on Chitosan Capped Gold Nanoparticles Modified Electrodes

**DOI:** 10.3390/bios2020189

**Published:** 2012-05-14

**Authors:** Aysu Yarman, Bettina Neumann, Maria Bosserdt, Nenad Gajovic-Eichelmann, Frieder W. Scheller

**Affiliations:** Fraunhofer Institute for Biomedical Engineering, IBMT, D-14476 Potsdam, Germany; E-Mails: aysu.yarman@yahoo.de (A.Y.); bettina.neumann1@web.de (B.N.); maria.bosserdt@ibmt.fraunhofer.de (M.B.); nenad.gajovic@ibmt.fraunhofer.de (N.G.-E.)

**Keywords:** peroxide dependent catalysis, hemin, microperoxidase-11, cytochrome c

## Abstract

In view of the role ascribed to the peroxidatic activity of degradation products of cytochrome c (cyt c) in the processes of apoptosis, we investigate the catalytic potential of heme and of the cyt c derived heme peptide MP-11 to catalyse the cathodic reduction of hydrogen peroxide and to oxidize aromatic compounds. In order to check whether cyt c has an enzymatic activity in the native state where the protein matrix should suppress the inherent peroxidatic activity of its heme prosthetic group, we applied a biocompatible immobilization matrix and very low concentrations of the co-substrate H_2_O_2_. The biocatalysts were entrapped on the surface of a glassy carbon electrode in a *biocompatible* chitosan layer which contained gold nanoparticles. The electrochemical signal for the peroxide reduction is generated by the redox conversion of the heme group, whilst a reaction product of the substrate oxidation is cathodically reduced in the substrate indication. The catalytic efficiency of microperoxidase-11 is sufficient for sensors indicating HRP substrates, e.g., p-aminophenol, paracetamol and catechol, but also the hydroxylation of aniline and dehalogenation of 4-fluoroaniline. The lower limit of detection for p-aminophenol is comparable to previously published papers with different enzyme systems. The peroxidatic activity of cyt c immobilized in the chitosan layer for catechol was found to be below 1 per mill and for p-aminophenol about 3% as compared with that of heme or MP-11.

## 1. Introduction

Cytochrome c is one of the best structurally characterized proteins. It was one of the first globular proteins whose crystal structure was determined. Its function in the respiratory chain as an electron transferase between complex (III) and (IV) is well understood on the molecular level. On the other hand, its role in the programmed cell death and in the early stages of neurodegeneration, such as Parkinson’s disease, is still the subject of investigations.

It has been frequently described that cyt c has no enzymatic activities in its native state because the protein matrix suppresses the inherent peroxidatic activity of its heme prosthetic group [1].

Iron protoporphyrine IX is the prosthetic group of the so-called heme proteins and heme peptides. (*Heme* is iron(II) protoporphyrine IX, whereas *hemin* contains iron(III)). Iron protoporphyrine IX has four pyrrole rings joined by methene bridges with an iron central atom. The two remaining coordination sites can be ligated by histidine, methionine or water in the different proteins.

Due to its high stability, its ability to mimic the reactions of heme proteins/enzymes and its low price, heme is also applied in sensor development. Furthermore, heme can be used as a catalyst for studying the *in vitro* drug metabolism [2].

Heme also plays an important role in diseases such as malaria. It is split off from hemoglobin (Hb) during plasmodium infection and interacts in the inflammatory reactions with reactive oxygen species (ROS) like superoxide and peroxide. In sickle cell hemoglobin no free heme is accumulated, since a heme degrading enzyme is induced. Therefore, sickle cell Hb prevents the outbreak of malaria.

Partial denaturation of cytochrome c by chemical reagents, like SDS [3], interaction with organic solvents [4] or ionic liquids [5] and binding to lipid membranes induces a peroxidatic activity. In addition, the treatment of cyt c with H_2_O_2_ in concentrations above 100 µM results in the generation of peroxidatic activity [6]. When cyt c is slightly perturbed, the 5th ligand methionine is displaced and the free 6th position is able to bind peroxide in a manner similar to heme peroxidases. Furthermore, the heme cavity gets accessible for water and substrate molecules which results in the generation of peroxidatic and/or catalatic activity of the heme protein.

It has been postulated that the cyt c-catalysed oxidation of cardiolipin and phosphatidylserine by ROS leads to permeabilization of the outer mitochondrial membrane for proapoptotic factors including cyt c. Once it is released into the cytosol, the binding of cytochrome c to the apoptosis protease activating factor (Apaf) initiates the activation of the caspases cascade [7,8].

For the early stage of neurodegeneration, it is postulated that cyt c is released from dysfunctional mitochondria into the cytosol where it is converted to heme peptides by cytosolic proteases. They may at least in part be responsible for the oxidative reactions of neurodegenerative diseases such as Parkinson’s disease, Alzheimer’s disease and amyotrophic lateral sclerosis.

The peroxidatic activity of unfolded cyt c is similar to that of microperoxidases which can be obtained by the proteolytic digestion of the parent protein. Furthermore, microperoxidases have P450-like activity and can catalyze sequential dehalogenation, hydroxylation and phenolic oxidation of halogen-substituted aromatic compounds like fluoraniline [9].

In view of the role that the peroxidatic activity of degradation products of cyt c may play in the processes of neurodegeneration or apoptosis, we investigate the potential of cyt c, of its prosthetic group hemin and of the cyt c derived heme peptide MP-11 to oxidize aromatic compounds, e.g., p-aminophenol, catechol, aniline and paracetamol. 

## 2. Experimental Section

### 2.1. Reagents

Cytochrome c from horse heart, microperoxidase-11 (MP-11) from horse heart cytochrome c were purchased from Sigma (Steinheim, Germany) and hemin from Fluka (Germany). 

Hydrogen peroxide (H_2_O_2_ 30%), HAuCl_4_, chitosan (85% deacetylated), catechol, paracetamol were purchased from Sigma (Steinheim, Germany) and p-aminophenol (pAP) from Fluka (Germany). 

All reagents were of analytical grade and used without further purification.

### 2.2. Preparation of Electrodes

Gold nanoparticles capped with chitosan (AuNP-CH) were prepared according to the method developed by Bonnard *et al*. Briefly, 80 µL of 4% of HAuCl_4_ acid solution in water were added to 50 mL distilled water containing 250 µL 0.2 M K_2_CO_3_ in ice bath and stirred continuously. 500 µL 0.5 mg/mL sodium borohydride solution were added stepwise and stirring continued for 20 min. 4 volumes of these particles were mixed with 1 volume of 1% (w/v) chitosan solution in 1% acetic acid [10].

The glassy carbon electrodes (GCEs) were polished with 1.0, 0.3 and 0.05 μm Al_2_O_3_, respectively and cleaned with Milli-pore water by sonication immediately before each use. 

Modification of the electrodes followed the method reported elsewhere [11]. Biocatalyst-AuNP-CH modified electrodes were prepared by dropping 10 µL of a 1:1 mixture of stock solution of biocatalyst and AuNP-CH solution onto the freshly polished GCE surface (Biocatalyst-AuNP-CH/GCE). Since cyt c is positively charged, a dialysis membrane (molecular cut off 10 kDa) was fixed over the chitosan layer to prevent its leakage from the electrode. For control experiments, electrodes without biocatalysts or without gold nanoparticles were prepared. All modified electrodes were dried at 4 °C overnight and rinsed thoroughly with Milli-pore water prior to use. 

### 2.3. Apparatus and Electrochemical Measurements

Electrochemical measurements were performed in a stirred electrochemical cell with a three-electrode configuration. A glassy carbon disk electrode (3 mm in diameter) was used as the working electrode, an Ag/AgCl (in 1 M KCl solution) electrode was the reference electrode, and a platinum wire served as counter electrode. 

Cyclic voltammetry (CV) and amperometry were performed using an Autolab PGStat 30 (Eco chemie, Netherlands). All experiments were carried out at room temperature (22–23 °C). Experiments for the direct electron transfer were performed in an anaerobic chamber (COY; USA). CVs were recorded in Sorensen buffer (10 mM, pH 5–7) or 10 mM phosphate buffer (pH 7) and scanned from −0.6 to +0.6 V with different scan rates. 

Amperometric measurements were performed under aerobic conditions. A working potential of 0 V or 0.02 V for the reduction of the product was applied. After 120 s under constant stirring, the background current reached a stable state. Once baseline stabilisation had occurred, 10 µM H_2_O_2_ were added. Current was recorded after stepwise addition of the substrate into the reaction chamber as a function of time.

## 3. Results and Discussion

### 3.1. Direct Electron Transfer

Direct electron transfer (DET) between the biocatalyst and the electrode surface is important for the characterization of the redox properties of the proteins or enzymes, but also for the development of the third generation of amperometric biosensors. According to the theory of Marcus and Sutin for the direct electron transfer of proteins, the rate constant of this direct electrochemical communication is governed by the potential difference between the involved redox centers, the reorganization energy, and most significantly, the distance between the protein and the electrode surface [12,13]. 

**Figure 1:**
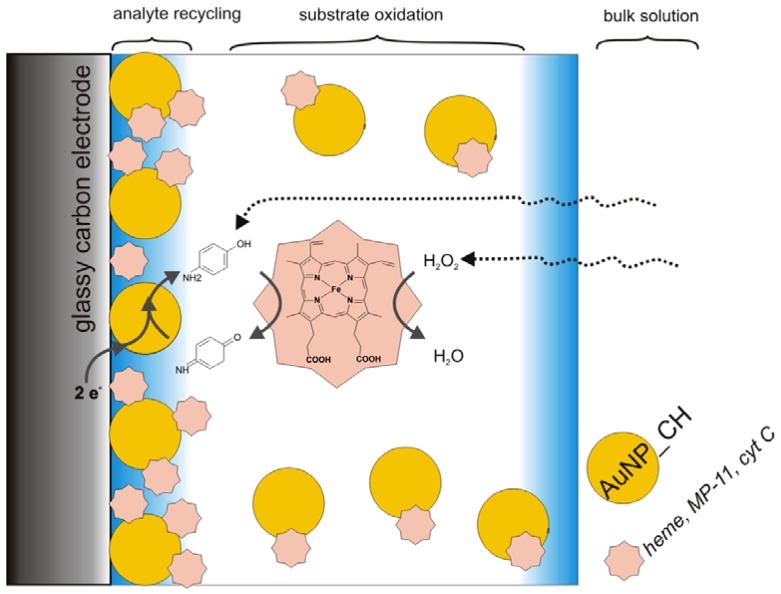
Schematic representation of the electrode.

In this work, the immobilization process is based on the electrostatic interaction between the positively charged matrix of chitosan (pK_a_ = 6.3) cappped AuNPs with negatively charged hemin or MP-11 (Figure 1). 

Recently we published data about direct electron transfer of MP-11 on AuNP-CH/GCE where chitosan capped gold nanoparticles were prepared in one step using chitosan as both reductant and stabilizer [14]. In this work the results are based on AuNPs which were prepared in two steps as described in the experimental section.

**Figure 2:**
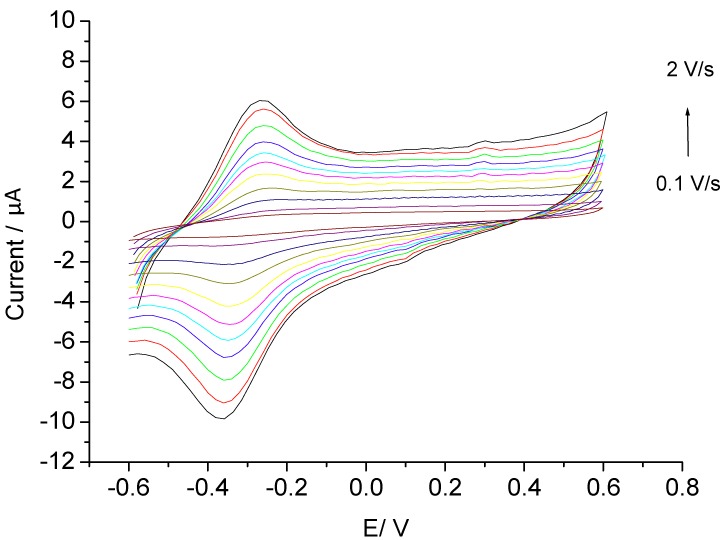
Cyclic voltammograms of Hemin-AuNP-CH/GCE at different scan rates.

Firstly, DET of hemin on the AuNP-CH modified electrodes was investigated. The cyclic voltammograms of Hemin-AuNP-CH/GCE (Figure 2) exhibit an anodic and a cathodic peak of almost equal heights. Both anodic and cathodic peak currents increase linearly with increasing scan rates between 0.1 and 2 V∙s^−1^, which indicates a surface-controlled electrode process. The peak separation (ΔEp) is 89 mV at 2∙V∙s^−1^ which is a characteristic of quasi-reversible systems.

The formal potential, *E^0^^′^*, of hemin in the gold nanoparticles-chitosan film was estimated to be −(307.9 ± 4.8) mV *vs.* Ag/AgCl electrode at pH 7.0, by taking the midpoint of anodic and cathodic peak potentials. This value is comparable with the value for hemin on carbon nanotubes modified GCE [15] but more negative than at chemically converted graphene on GCE (−160 mV). On the other hand, it is more positive than previously published data for the Fe^2+^/Fe^3+^ redox couple of hemin such as −374 mV (Ag/AgCl) on GCE [16], −380 mV (*vs.* Ag/AgCl) on PGE [17], −390 mV (*vs.* Ag/AgCl) on carbon paste electrode [18] and −380 ± 10 mV (*vs.* SCE) on surface of the Fe_3_O_4_@SiO_2_ particles [19].

The surface coverage (Γ) of the electrochemically active hemin was determined to be 386.9 pmol∙cm^−2^ at 100 mV/s from the integration of the anodic peak, by using the equation Γ = Q/nFA, where n is the number of electrons transferred during the redox process, F is the Faraday constant and A is the area of the electrode (7.07 mm^2^). Assuming that the cross section of one heme molecule is 2.38 nm^2^, the surface concentration of a monolayer at a flat electrode should be 70 pmol∙cm^−2^. Thus in the present work, more than one monolayer was observed. In an earlier paper [17] for hemin adsorbed on a pyrolytic graphite electrode, a surface concentration of 450 pmol∙cm^−2^ of the “real surface” was found.

The heterogeneous rate constants (k_s_) of hemin adsorbed at the AuNP-CH/GCE was determined by Laviron’s method [20]. The value for k_s_ starts at 0.88 s^−1^ and levels off at 23.74 s^−1^ in the scan rate range from 0.1 to 2.0 V∙s^−1^. Higher rate constants such as 4,000 s^−1^[21] when heme was adsorbed on basal pyrolytic graphite or GC electrodes, or 3,600 s^−1^ on mercaptoethanol modified gold electrode were obtained. The lower rate constant obtained by this study might be caused by the multilayer electron transfer process. 

**Figure 3:**
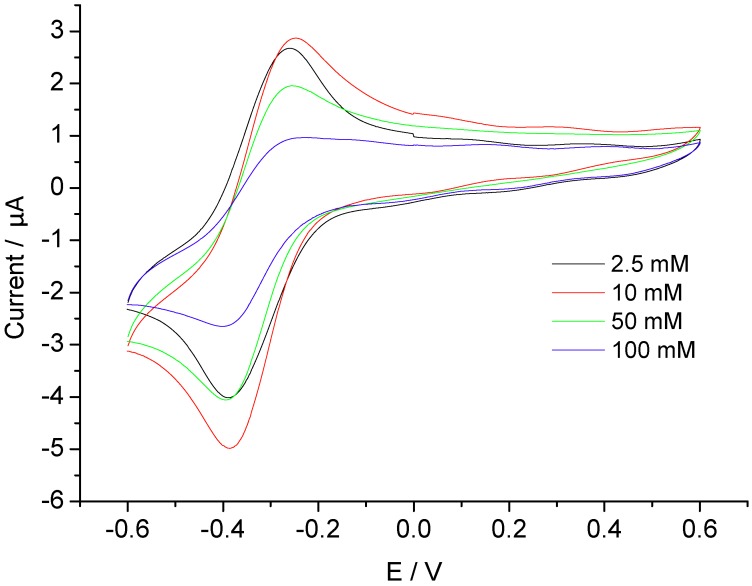
Cyclic voltammograms of MP-11-AuNP-CH/GCE in different buffer concentrations at pH 7.

Furthermore, we investigated the DET of MP-11 at the AuNP-CH modified GC electrodes and elucidated the effect of ionic strength of the measuring buffer solutions. MP-11 displayed in the cyclic voltammograms a pair of quasi-reversible redox peaks at all ionic strength, whilst in the absence of MP-11 the AuNP-CH modified GCE did not show a redox signal (not shown). As shown in Figure 3, the peak currents decrease with increasing ionic strength between 2.5 mM to 100 mM at pH 7. The formal potential of MP-11 in 10 mM PB, at pH 7 was calculated to be −335.75 mV at the scan rate of 100 mV/s. 

**Figure 4:**
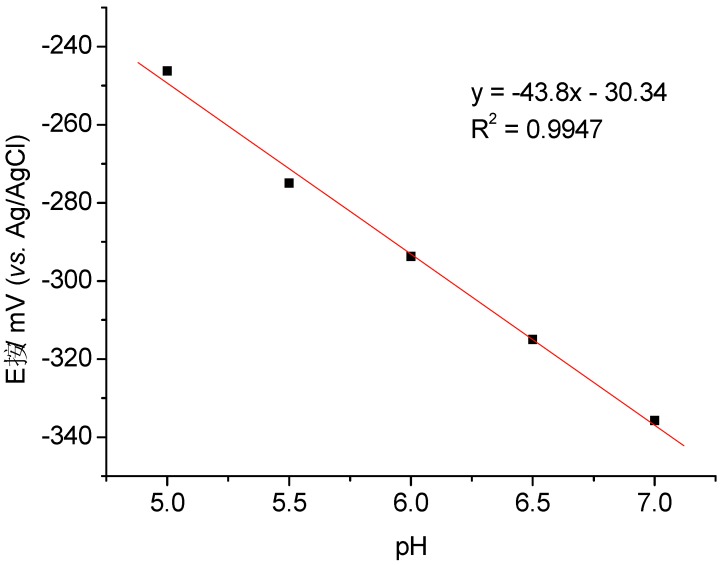
Formal potentials of MP-11 immobilized in a matrix of chitosan embedded AuNPs at GCE at pHs between 5 and 7 at 100 mV/s.

Figure 4 shows the effect of pH in 10 mM phosphate buffer on the DET of MP-11-AuNP-CH/GCE at 100 mV/s. The formal potentials (*E^0’^*) of MP-11 in the gold nanoparticles-chitosan film were determined in the pH interval between 5 and 7. They are shifted in anodic direction by almost 89 mV at pH 5 as compared with the value at pH 7. This behavior is in accordance with the pH dependence of the redox potential of microperoxidases in the literature which ascribes the potential shift at pH 5 to the dissociation of the propionic side chain of the heme [22]. The slope of the line dE/dpH was calculated to be −43.8 which is close to one electron/ one proton transfer. The positions of peaks and of the formal potentials are comparable to previously published data for the Fe^2+^/Fe^3+^ redox couple of MPs [23,24].

We determined the “electrochemically active” surface concentration in order to compare our arrangement with the literature and to quantify the “excess” of MP in relation to the monolayer approach. The electrochemically active surface concentration of MP-11 on chitosan capped AuNPs at pH 7 was determined to be 371.3 pmol∙cm^−2^ at a scan rate of 100 mV/s. For adsorbed MP-11 on alkanthiol modified gold electrodes the surface concentration of a monolayer was reported to be in the range from 17 to 54 pmol∙cm^−2^ [23]. Taking into account a roughness factor of 1.5 to 3.0, a dense monolayer of MP-11 at the glassy carbon surface should be between 90 and 180 pmol∙cm^−2^ [14]. The calculated value reflects the ability of chitosan embedded AuNPs to increase the electrode active surface area for MP-11 adsorption and only AuNPs in the intimate vicinity of the electrode are involved in the electrode process.

The heterogeneous rate constants (k_s_) for MP-11 at AuNP-CH/GCE levelled off at 12 s^−1^ between pH 5 and 7 at 2 V/s. The value of k_s_ is in the range for multilayers of MP-11 [23]. However, it is considerably smaller than for monolayers for MP-11 where values up to 1,900 s^−1^ have been reported [23].

In contrast to hemin and MP-11, DET for cyt c modified AuNP-CH/GCE could not be obtained probably due to the lack of correct orientation of the heme group of the protein towards the electrode. However, Feng *et al*. obtained DET of cyt c on chitosan capped AuNPs electrode by using cysteine. The *E^0’^* was calculated to be 185 mV *vs.* SCE which is almost 170 mV more positive than at mercaptoundecanoic acid modified gold electrodes [25]. 

### 3.2. Catalysis of Cathodic Reduction of Peroxide

The product of the reaction between heme or microperoxidase and hydrogen peroxide is an analogue to the HRP-Compound I which is two oxidizing equivalents above the ferric form with Fe(IV) and a π-cation porphyrine radical. This species has been identified by UV-VIS and resonance Raman spectroscopy [26]. The reaction with hydrogen peroxide to the reactive species is for MPs almost three orders of magnitude slower than for HRP [26]. The formal potential of the reaction Compound I/Compound II has been determined for HRP to be more positive than 650 mV *vs.* SCE [27].

The high formal potential of the Compound I analogue should lead at MP-modified electrodes to formation of the cathodic currents starting around 600 mV *vs.* SCE. In several papers for carbon, gold, tin oxide, platinium and ITO electrodes the cathodic reduction starts between 200 and 400 mV *vs.* SCE (Table 1). This behavior has been ascribed to a combination of direct electron transfer from Compound I and the action of redox active groups at the electrode surface as mediators. In fact, the pretreatment of the electrode surface has a large effect on the catalytic peroxide reduction. On the other hand, there is a large number of reports describing the peroxide reduction at the potential where the heme is in the reduced state. In analogy to unfolded cytochrome c [28] also for microperoxidase the reaction with peroxide could lead via Fe(II) to Fe(IV) = O [29]. This species has a remarkably more positive formal potential than the ferric heme and it is immediately reduced at this potential along with the reduction of ferric microperoxidase. As an alternative to the previous mechanism, the formation of OH^.^radicals in a Fenton type reaction between Fe(II) and peroxide has been postulated, since cumarine was hydroxylated to a fluorescent product [30] by MP-11 immobilized on a mesoporous tin oxide electrode. This highly active species has the ability to perform reactions in either peroxidatic or a P450 mode [22]. 

**Table 1:** Peroxide catalysis by MP-11 modified electrodes.

Electrode preparation	Linear range (µM)	LOD	K_M_	Reference
MP-11-CPE			55(±12) mM	[31]
MP-11-HCPE	-	-	6.4(±1.1) mM	
MP-8-polypyrrole	1–10	10 nM		[32]
MP-11-chitosan–graphene-nanocomposite	2.5–135	2 µM	0.54 mM	[24]
MP-11-MWNT/GC	-	-	2.4 mM	[33]
MP-11-GNP-MWNT/GC	10–200	3 µM	0.32 mM	[34]
MP/ZnO NPs/PG	1–700	30 µM	-	[35]
MP-11-DDAB/GC	2.4 mM and 20 µM	0.8 µM	-	[36]
(MP11/PNTs/PAH)_n = 4_/ITO	-	6 µM	-	[37]
MP-11/RW	30 µM-4 mM		5–10 mM	[27]
MP-11-AuNP-CH/GC	1–7	0.27 µM	4.43 µM	[14]
MP-11-MWNT	5–70	3.8 pM		[38]
MP-11-Nanopolyurethane/GC	0.02–1.3	10 pM	(1.87 ± 0.05) µM	[39]
MP-11-SnO_2_-PLL	0.05–30	50 nM		[30]

MWNT: multi-wall carbon nanotubes, PG: pyrolytic graphite, PLL: poly-L-lysine; GNP: Gold nanoparticle.

The cyt c-AuNP-CH/GCE did not generate a catalytic current on addition of peroxide. On the other hand, urea-unfolded yeast cyt c electrostatically adsorbed at an anionic self-assembled monolayer on a gold electrode yields electrocatalytic reduction of peroxide. The cathodic peak potential of −300 mV does not change with increasing peroxide concentration which indicates that the reduced state of cyt c reacts with the cosubstrate. The concentration dependence follows the Michaelis-Menten equation with a K_M_ value of around 10 µM. This value is three orders of magnitude smaller than that for cyt c immobilized on modified electrodes [40,41]. For peroxide concentrations above 40 µM, the catalytic current irreversibly decreases [28].

### 3.2. Peroxide-Dependent Substrate Oxidation by Hemin, Microperoxidase-11, and Cytochrome C

Metalloporphyrins including hemin and microperoxidases are able to catalyse a spectrum of reactions, e.g., phenolic oxidation, epoxidation, aromatic hydroxylation or oxidation of heteroatoms [2]. As early as 1975 we described the peroxidatic activity of ligated hemin using the substrates pyrogallol and ascorbic acid [42]. In order to suppress the oxidative degradation of the porphyrin derivatives carrying electron, withdrawing or sterically hindered substituents have been developed [2].

In analogy to the oxidation of phenols by HRP, also Compound I of hemin and microperoxidases follows a ping pong mechanism with two successive one-electron steps. However, the HRP analogous pathway is accompanied by irreversible degradation of the porphryrin by the attack of peroxide. The cleavage of the O-O bond of peroxide occurs heterolytically and OH^.^ radicals should not be involved. This has been concluded from the ineffectivity of radical scavengers like formate and mannitol [22].

The oxidation of phenolic substrates (hydroquinone, *p*-aminophenol, paracetamol, and resorcinol) is based on one-electron/hydrogen abstractions leading to reactive phenoxyl radicals that tend to couple and polymerize or to form benzoquinones and quinoneimines, respectively by disproportionation [43]. The oxidation of phenolic substrates in this way is a characteristic reaction of heme peroxidases. 

The conversion of aniline into hydroxylaminobenzene and aminophenols represents two-electron oxidation associated with an oxygen transfer from the peroxide to the product (P450-like reaction). These reactions are not inhibited by ascorbic acid [44].

**Figure 5:**
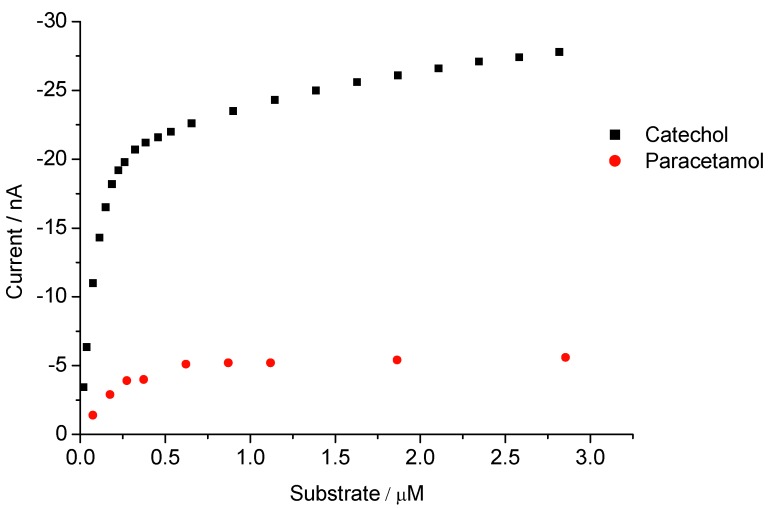
Concentration dependence for catechol and paracetamol at 0 V of the Hemin-AuNP-CH/GCE.

In the present work hemin-based sensors for catechol, paracetamol and aniline were developed. As seen from Figure 5, in the presence of 10 µM peroxide, the current for the reduction of the reaction product is linearly dependent between 18.7 and 186 nM for catechol and for paracetamol from 75 to 275 nM, respectively. In the case of catechol, the linear range extends up to 458 µM and the signal is almost 3.4 times higher in the presence of l mM peroxide instead of 10 µM (data not shown). 

**Figure 6:**
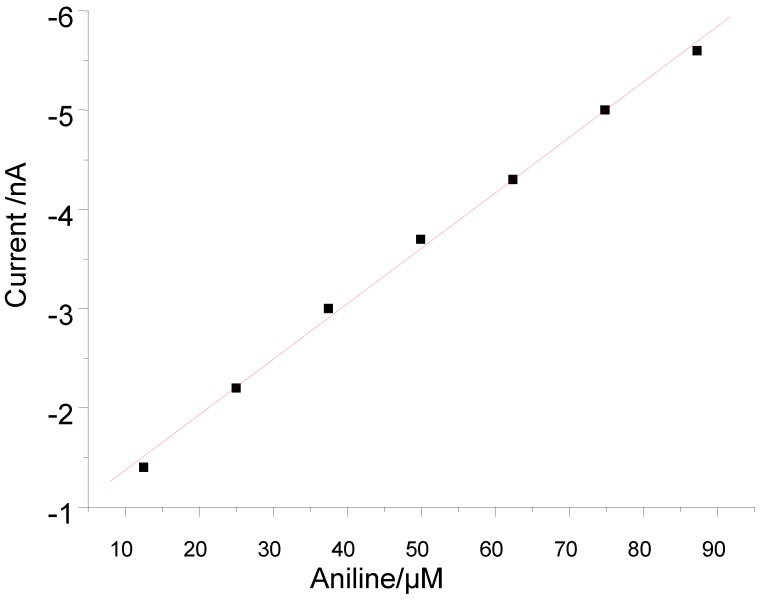
Concentration dependence for aniline at 0 V of the Hemin-AuNP-CH/GCE.

In contrast to catechol and paracetamol with hemin based sensor, for aniline no response was obtained in the lower micromolar range, but the current increased linearly between 124.9 and 871 µM on stepwise addition of aniline in the presence of 10 µM peroxide (Figure 6).

**Figure 7:**
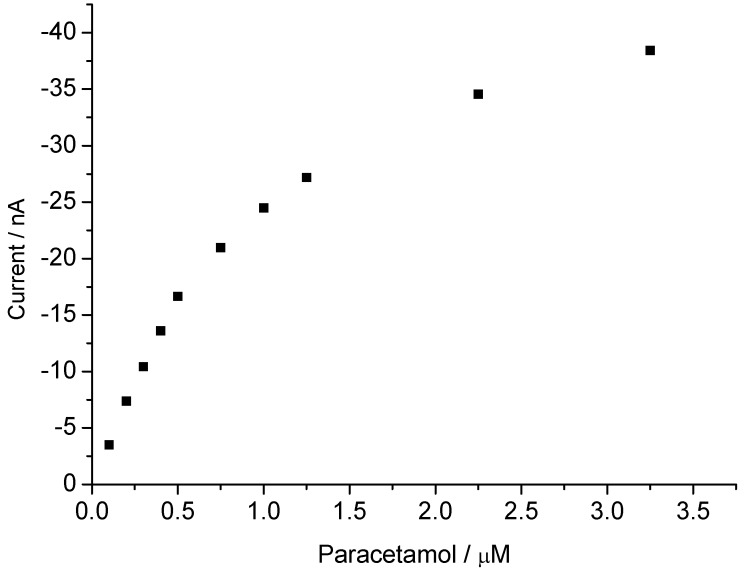
Concentration dependence for paracetamol at 0 V of the MP-11-AuNP-CH/GCE.

For the sensor using microperoxidase in the chitosan AuNP immobilization matrix, for *p*-aminophenol and catechol we obtained almost identical sensitivities which were in the linear range twofold and, at saturation, 12-fold higher than for aniline. No response toward *p*-nitrophenol was found [9]. The current-concentration dependence for paracetamol is shown in Figure 7. The current increases linearly between 0.1 to 1 µM on stepwise addition of paracetamol in the presence 10 µM peroxide. 

In order to prevent the destruction of heme by peroxide, we applied a low concentration of peroxide. To check the effect of higher concentration of the cosubstrate peroxide, we did successive measurements with the same electrode starting with 10 µM up to 100 µM (). The amperometric responses for the same concentration of pAP did not change significantly with the increase of peroxide concentration. However, after addition of 100 µM peroxide, the following measurement with 10 µM peroxide showed a decrease of the response by 30% as compared with the first measurement with 10 µM peroxide.

**Figure 8:**
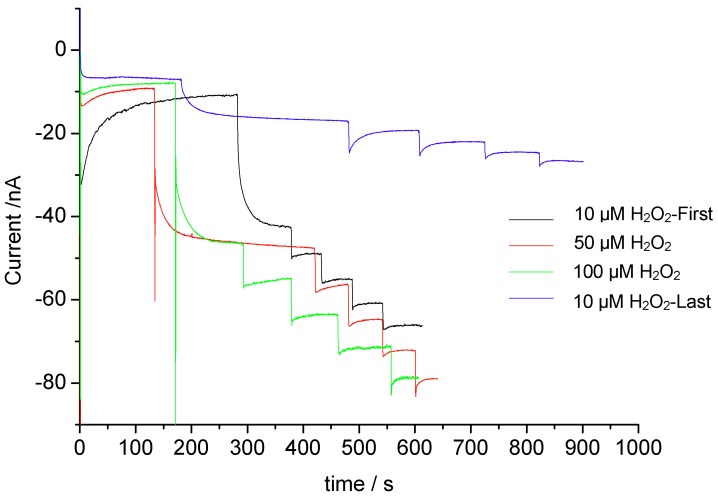
Amperometric responses of MP-11-AuNP-CH/GCE on stepwise addition of pAP in presence of different peroxide concentrations.

The catalytic activity of microperoxidases is sufficient at 10 µm peroxide for the signal generation/oxidation of HRP substrates, e.g., pAP, catechols or ascorbic acid, but also the hydroxylation of aniline and dehalogenation of 4-fluoroaniline. The lower limit of detection for pAP is almost 70 times lower than for the direct anodic oxidation of pAP [45] and comparable to previously published papers with different enzyme systems which exploit bi-enzymatic or electro-enzymatic signal amplification [46,47,48,49].

In our previous paper we circumvented the problems of low specific activity and the destruction of the heme group by peroxide by the almost hundred fold excess of MP as compared with a complete monolayer and signal amplification by electro-enzymatic analyte recycling [9]. 

As compared with HRP, the MP-11 based electrode has the advantage that aromatic substances like aniline but also the drug diclofenac are hydroxylated. These findings will enable us to develop sensors for the determination of drugs, e.g., diclofenac, and anthraquinone derivatives.

Futhermore, we found that cyt c immobilized in the chitosan layer exhibits a negligible peroxidatic activity for the phenolic oxidation of catechol at µmolar concentrations of the co-substrate peroxide. It is less than one per mill as compared with that of heme or MP-11. Also, for the peroxide-dependent conversion of aniline we found no sensor signal above the background noise. For p-aminophenol the electrochemical signal for the oxidation product is between 1 and 3 percent as compared with that of MP-11. This residual activity may be an intrinsic property of native cyt c or it might be caused by a minimal perturbation of the protein.

Schmidt *et al*. compared the catalytic activity of HRP, myoglobin (Mb), MP-11 and cyt c in the peroxide-dependent reaction of 4-aminoantipyrine with phenol. MP-11 and Mb exhibit almost 1 per mill of the activity of HRP and the value for cyt c is even five times lower than that of microperoxidase. They ascribed the peroxidatic activity to the “partial denaturation” of cyt c by 5 mM peroxide. In accordance with Lötzbeyer [13] we found for the peroxide-dependent oxidation of catechol by hemin, a 3–4 fold higher signal than for MP-11. However, the cyt c sensor showed a response which was two to three orders of magnitude smaller than for hemin or MP-11 and not only five times as described by Lötzbeyer *et al*. 

Dong *et al*. [50] reported that m-aminophenol -a (inhibiting) type II substrate of P450- is hydroxylated and subsequently dimerized in the peroxide dependent catalysis by cyt c. Surprisingly, an excess of the radical scavengers mannitol or benzoate completely suppress the product formation.

Similarly, phenol is oxidized to diphenoquinone only in the presence of (electrochemically) reduced cyt c and peroxide, but not by the oxidized protein [51]. 

Free and immobilized cyt c catalyses in aqueous mixtures of organic solvents like tetrahydrofuran, acetonitrile, the peroxide-dependent N- and O-demethylation, S-oxidation, epoxidation of olefins and P450-like hydroxylations. Superoxide and hydroxyl radicals are not involved in these reactions, since superoxide dismutase or mannitol are inactive [4]. 

## 4. Conclusions

We report in this paper that the enzyme electrodes, using heme, MP-11 or cyt c, immobilized in a chitosan layer at lower µmolar concentrations of the cosubstrate peroxide, exhibit quite different analytical characteristics. Whilst heme converted catechol considerably more efficiently than aminophenol, the MP-11 based sensor exhibited almost identical sensitivity towards both substrates. The peroxidatic activity of cyt c for catechol is almost negligible and for p-aminophenol only between 1 and 3 percent as compared with that of its prosthetic group heme or the heme peptide MP-11. These differences might allow for a differentiation between cytochrome c and heme peptides.

The residual activity found in our studies may be an intrinsic property of native cyt c, or it is caused by a minimal perturbation of the protein. The induction of the pronounced peroxidatic activity in the early stage of apoptosis or neurodegeneration obviously requires a pronounced perturbation of the protein; probably by enhanced peroxide concentrations.
